# Impact of a Smart-Ring-Based Feedback System on the Quality of Chest Compressions in Adult Cardiac Arrest: A Randomized Preliminary Study

**DOI:** 10.3390/ijerph18105408

**Published:** 2021-05-19

**Authors:** Chiwon Ahn, Seungjae Lee, Jongshill Lee, Jaehoon Oh, Yeongtak Song, In Young Kim, Hyunggoo Kang

**Affiliations:** 1 Department of Emergency Medicine, College of Medicine, Chung-Ang University, Seoul 06974, Korea; cahn@cau.ac.kr; 2 Department of Biomedical Engineering, Hanyang University, Seoul 04763, Korea; seungjaelee@hanyang.ac.kr (S.L.); iykim@hanyang.ac.kr (I.Y.K.); 3 Department of Emergency Medicine, College of Medicine, Hanyang University, Seoul 04763, Korea; ojjai@hanyang.ac.kr (J.O.); yeongtaksong@hanyang.ac.kr (Y.S.); emer0905@gmail.com (H.K.); 4 Convergence Technology Center for Disaster Preparedness, Hanyang University, Seoul 04763, Korea

**Keywords:** cardiopulmonary resuscitation, heart arrest, smart-ring, chest compression, feedback, simulation

## Abstract

This study aimed to assess the effectiveness of a novel chest compression (CC) smart-ring-based feedback system in a manikin simulation. In this randomized, crossover, controlled study, we evaluated the effect of smart-ring CC feedback on cardiopulmonary resuscitation (CPR). The learnability and usability of the tool were evaluated with the System Usability Scale (SUS). Participants were divided into two groups and each performed CCs with and without feedback 2 weeks apart, using different orders. The primary outcome was compression depth; the proportion of accurate-depth (5–6 cm) CCs, CC rate, and the proportion of complete CCs (≤1 cm of residual leaning) were assessed additionally. The feedback group and the non-feedback group showed significant differences in compression depth (52.1 (46.3–54.8) vs. 47.1 (40.5–49.9) mm, *p* = 0.021). The proportion of accurate-depth CCs was significantly higher in the interventional than in the control condition (88.7 (30.0–99.1) vs. 22.6 (0.0–58.5%), *p* = 0.033). The mean SUS score was 83.9 ± 8.7 points. The acceptability ranges were ‘acceptable’, and the adjective rating was ‘excellent’. CCs with smart-ring feedback could help achieve the ideal range of depth during CPR. The smart-ring may be a valuable source of CPR feedback.

## 1. Introduction

Sudden cardiac arrest is a leading cause of death in developed and developing countries [1,2,3]. In addition, the loss of physical function and an increase in medical costs because of cardiac arrest are recognized as important public health problems worldwide [4,5]. Accordingly, it is important that cardiopulmonary resuscitation (CPR) is provided accurately and effectively to improve patient outcomes. The latest American Heart Association guidelines for CPR recommend a chest compression (CC) depth of 5–6 cm and a compression rate of 100–120 times per minute in adult patients [6]. Moreover, the guidelines state that audio-visual feedback may help achieve efficient CCs [6]. A pressure or an acceleration sensor in a feedback device can recognize the depth and speed of CCs during CPR and provide audio–visual feedback and guidance. Previous systematic reviews and meta-analyses of studies on CPR feedback device use have shown that significant improvements in parameters such as CC depth, rate, and accuracy occur with device use [7,8].

After an increase in the penetration rate of smart devices, development and research of a feedback system for CCs using the acceleration sensor in smart devices have also increased [9,10,11,12,13,14]. Some evidence suggests that feedback systems incorporated into smartphones or smartwatches may partly or wholly help improve the accuracy, depth, and rate of CCs [9,10,11,12,13,14,15,16]. Previously, we presented a system that uses an acceleration sensor to provide color-coded visual feedback, which captures the CC depth on a smartwatch screen [17,18]; this system was tested in a cardiac arrest model, using a manikin [14]. In that study, we showed that smartwatch-based feedback may help improve the accuracy of CC depth [14].

However, this kind of device comes with limitations associated with the position of the watch, which is placed on the wrist, where visual feedback is difficult to assess. In addition, a watch may cause pain when pressed into the back of the hand during compressions [19]. To improve this device and the accuracy of the CC algorithm, we developed a ring-type feedback device [19], operated by a new algorithm [19]. In this study, we conducted an adult cardiac arrest manikin-based simulation experiment with the smart-ring device, and CC-related parameters were assessed. In addition, the usability and learnability of this new smart-ring were evaluated with the System Usability Scale (SUS) score.

## 2. Materials and Methods

### 2.1. Design

This randomized study was designed as a prospective crossover study. We investigated the impact of a new feedback algorithm in a smart-ring device on the quality of CCs administered during a simulation of adult cardiac arrest. This study was conducted at the Simulation Center of Hanyang University in January 2020. We registered the study protocol in the Clinical Research Information Service (KCT0005559).

### 2.2. Participants

Twenty volunteers participated. The eligibility inclusion criteria were healthy persons over 18 years and having undergone at least one basic life support training program. Participants with wrist pain, back pain, or disease of the lung/heart were excluded. They reviewed and approved the contents of this study and provided written informed consent.

### 2.3. Equipment and Materials

We used the Skill Reporter™ manikin (Laerdal, Stavanger, Norway), and a dedicated program that was used for the recording was operated on a laptop during the simulation. The program estimated the CC depth/decompression depth and compression rate through a mounted sensor. This program responded as the manikin’s central chest was being compressed. We used the feedback system of a smart-ring, which was previously developed and validated by our group [19]. This smart-ring device calculates the CC depth in real-time based on inertial measurement units and by considering the orientation of the device; it then provides feedback using a light-emitting diode (LED). The experiment was conducted on a flat and firm floor. 

**Figure 1:**
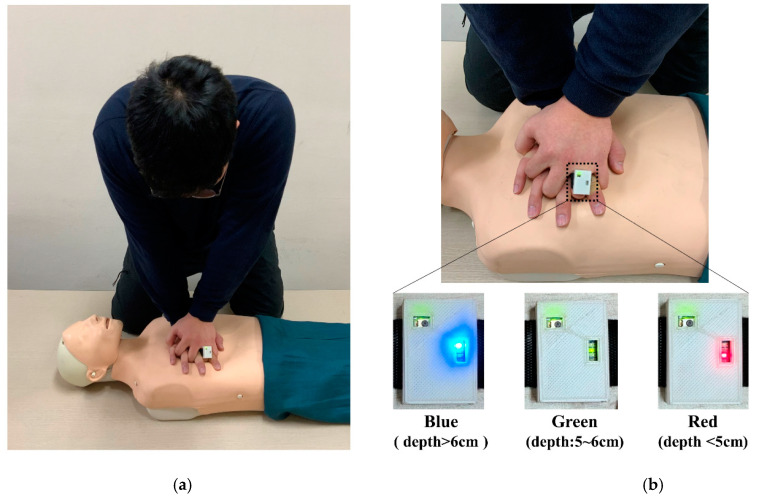
Visual feedback of the smart-ring device. (**a**) CPR posture and environment during chest compression. (**b**) Three LED lights were linked to the depth.

The participants performed CCs on a manikin while using the smart-ring feedback. Three LED light colors were linked to the compression depth (Figure 1). The blue, red, and green lights were presented at a CC depth of more than 6 cm, less than 5 cm, and 5 to 6 cm, respectively (Figure 1). These conditions were consistent with those of previous studies [14].

### 2.4. Grouping and Experimental Conditions

All participants were randomly allocated to two groups in a 1:1 ratio (group A and group B). Group A conducted an intervention experiment, followed by a control experiment 2 weeks later. Group B conducted the control experiment, followed by the intervention experiment 2 weeks later. The intervention condition involved the participants initiating CCs while using the smart-ring, which was running the feedback program. Compression was continued, except for rescue breathing, while in a kneeling position next to the manikin, for 3 min. Control experiments were conducted within the same parameters, except for the fact that no feedback device was used.

In order to blind the investigators to the group assignment, an invisible screen was placed between the investigators and the participants. Participant characteristics including the number of CPR training sessions attended and actual CPR experience were collected. Experimental records were extracted from the recording program of the manikin by one investigator who was blinded to the group allocation of the experiment.

### 2.5. Outcomes

The primary outcome was CC depth. For the additional outcomes—the proportion of accurate-depth CCs, CC rate, and the proportion of complete chest decompression—data were collected. The proportion of accurate-depth CCs was defined as the ratio of the number of CCs in which the depth was 5 to 6 cm to the total number of CCs. The proportion of complete chest decompression was defined as the ratio of the number of decompressions wherein the relaxation depth was less than 1 cm to the total number.

### 2.6. Learnability and Usability of the Smart-Ring 

For learnability and usability evaluations, we used the modified SUS, a simple but reliable method for evaluating the usability of a technological product or service [20,21,22]. The SUS consists of 10 statements: five positively worded statements (odd-numbered domain) and five negatively worded statements (even-numbered domain) as follows:

1. I would use this product frequently;
2. I think the product is unnecessarily complex;
3. I think the product is easy to use;
4. I think I would need technical support to learn how to use this product;
5. I think that the functions in the product are well-integrated;
6. I think there was much inconsistency in the performance of the product;
7. I imagine that most people would learn to use this product quickly;
8. I find the product very uncomfortable to use;
9. I feel confident using this product;
10. I will need to learn many things before continuing to use the product.

Statements (4) and (10) represent the value of learnability for laypersons, while the other statements represent the value of usability. The SUS uses five-scale domains numbered from 1 (strongly disagree) to 5 (strongly agree). To obtain a score, the following formulas were used:

- Positively worded domains = (score − 1);
- Negatively worded domains = (5 − score);
- After summing up the scores for the 10 domains, we multiplied by 2.5 = total SUS.

The acceptable range of the total score was assessed according to Bangor’s SUS criteria [21] (Supplementary Figure S1).

### 2.7. Statistical Analyses

The experimental records were analyzed using Excel 2016 (Microsoft, Redmond, WA, USA) and R (version 4.0.0, The R Foundation for Statistical Computing). Descriptive statistics were applied to express the general characteristics. Normally distributed variables were reported as mean ± standard deviation with 95% confidence intervals (CI), whereas non-normally distributed variables were reported as medians with interquartile ranges and 95% CI. The Kolmogorov-Smirnov test was used to test for the normality of data distribution in all datasets. A Wilcoxon signed rank sum test, which is a nonparametric method, was used to compare continuous variables. A multiple linear regression analysis was performed to identify factors that influenced the outcomes and to compare effect sizes. A Friedman test was used to compare the average value of CC depth in each of the two groups over the compression time. *p*-values of <0.05 were considered indicative of a statistically significant finding. 

## 3. Results

### 3.1. Group Allocation and Participant Characteristics

**Figure 2:**
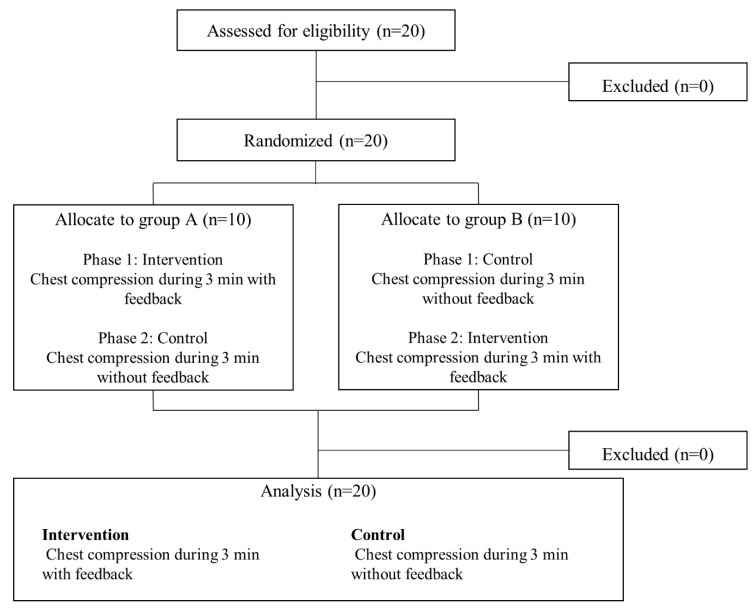
A flowchart of participant enrollment process.

A total of 20 volunteers (15 men and 5 women) participated in this experiment (Table 1). The participants’ median (interquartile range) number of CPR training sessions attended was 3 (2–3); none of the participants had real-world CPR experience. Group assignment is presented in Figure 2. Data of the 20 cases in each group were analyzed. 

### 3.2. Main Outcome 

Compression depth was 52.1 (46.3–54.8) mm in the intervention condition, which was significantly higher than that in the control condition (47.1 (40.5–49.9) mm) (*p* = 0.021). The proportion of accurate-depth CCs in the intervention condition was significantly higher than that in the control condition (88.7 (30.0–99.1) vs. 22.6 (0.0–58.5%), *p* = 0.033) (Table 2). Logistic regression models revealed a significant correlation between sex and body mass index and feedback system status, and between sex and body mass index and CC depth and the proportion of accurate-depth CCs (Table 3). 

### 3.3. Results of the Learnability and Usability Evaluation 

SUS scores for the smart-ring were investigated. Higher scores represented more positive evaluations of the smart-ring. The mean overall evaluation score was 83.9 (8.7) points. The mean usability score was 16.8 (2.7) points, and the mean learnability score was 67.3 (7.7) points. The acceptability ranges were evaluated as ‘acceptable’, and the adjective rating was ‘excellent’.

Supplementary Table S1 shows the converted item scores for each item. There were negative questions for questions 2, 4, 6, 8, and 10. Through conversion, the higher the score, the more positively it was interpreted. Questions 4 and 10 were items on learnability, while the other items were for usability. Among them, item 2 was evaluated as the highest (score 3.8), and the item on discomfort in item 8 also had a high conversion score (score 3.65).

## 4. Discussion

The feedback system of the ring-shaped smart device has not been reported on in previous studies on smart device feedback systems, and it is important to note that through this study, its clinical usefulness has been confirmed. The experimental group that performed CCs with feedback showed significantly better compression depth compared to the control group that performed CCs without receiving feedback. Furthermore, the experimental group also showed a significantly better proportion of accurate-depth CCs (CC depth with feedback 52.1 (46.3–54.8) mm, without feedback 47.1 (40.5–49.9), and the proportion of accurate CC depth (with feedback, 88.7 (30.0–99.1); without feedback, 22.6 (0.0–58.5)) than the latter group. These findings suggest that the smart-ring may help achieve desirable CCs by providing feedbacks during CPR.

CPR feedback systems using various smart devices have been studied over the past decade. A previous meta-analysis [23] included 11 such studies; three of them involved smartwatch-based feedback [14,15,16]. For example, Park showed that smartphone-based feedback did not improve the quality of CPR and that the hand pain associated with holding and operating a smartphone was a reason for complaint [24]. A smartwatch is a lightweight, wearable device that may overcome the limitations of smartphones; in fact, three previous studies have shown that it may help improve CC parameters [14,15,16]. Similarly, the smart-ring is compact, lightweight, and convenient to wear, and is associated with improved CC depth and an improved proportion of accurate-depth CCs. These findings suggest that it performs well as a feedback device. Moreover, the participants’ SUS evaluations have shown that this device is acceptable to users.

An advantage of the smart-ring is that it is placed on the finger. Error in compression depth estimates varies according to the position of the feedback device when using a smartphone [25], and since the smart-ring is placed near the sternum, it is relatively more likely to provide accurate feedback. However, in the present adult cardiac arrest simulation experiment, the positional advantage of the smart-ring sensor was not clear. When the palm of the hand is pressed against the sternum for CC, the position of the wrist with a smartwatch and that of the finger with a smart-ring is within a similar range. However, the advantage of the sensor location is expected to be relatively greater in infant CPR. 

In infants, CCs are performed using fingers, which contrasts with the method used in adult CPR. Pediatric CPR methods include two-finger or two-thumb encircling techniques [26]. CPR feedback from a device may help in infant cardiac arrest [26]; in fact, previous studies have shown that the depth of CCs on infants is significantly improved by using smartwatch-based feedback [16]. However, when performing CCs on infants with conventional feedback devices, the accuracy of the feedback may decrease as the acceleration sensor is moved away from the sternum by the length of the rescuer’s finger. The smart-ring can be positioned close to the sternum and may improve the feedback accuracy; however, future studies are required to verify this claim. In addition, a simulation study of smart-ring feedback in infants may be required.

This study had several limitations, which should be considered when interpreting its findings. First, this was a simulation study using a manikin; thus, real-world environmental and situational factors were not considered. Second, this feedback system with an accelerometer cannot compensate for the effect of mattress compression, and may be unsuitable to use for in-hospital bed-bound cardiac arrest patients. Third, this study used a crossover design, and the second experiment was conducted after a 2-week washout period to counteract the effect of learning. However, this design does not eliminate other sources of bias. Other changes—for example, an increase in muscle strength or endurance—may occur over the period of 2 weeks, affecting the participants’ performance in a CPR experiment. Fourth, although all participants had performed CPR at least once in the past, the quality of this intervention was not assessed. Fifth, this study had a gender imbalance in the participants involved. Thus, the possibility of bias in deriving the result could not be eliminated. Finally, the time required to activate the feedback program in the event of a cardiac arrest is important; however, it was not evaluated in this study.

## 5. Conclusions

CPR feedback from the smart-ring may help achieve the CC depth recommended in the American Heart Association CPR guidelines. The present findings suggest that the smart-ring feedback system achieves similar CC parameters to a previously proposed smartwatch-based system. This new smart device may help perform CCs effectively and efficiently. 

## Figures and Tables

**Table 1:** Baseline characteristics of the study participants.

Characteristics	Population		*p*-Value *
	Male (n = 15)	Female (n = 5)	
Age, years	23 (21.5–24.5)	21 (21–22)	0.356
Height, cm	172.0 (170.0–176.0)	162.0 (158.0–164.0)	0.001
Weight, kg	72.0 (65.5–79.0)	54.0 (53.0–55.0)	0.001
Body mass index	24.1 (21.9–27.0)	20.2 (20.0–21.6)	0.019
Number of CPR training sessions	3 (2–3)	3 (3–4)	0.558
Performance of CPR in real world	0 (0–0)	0 (0–0)	-

CPR (cardiopulmonary resuscitation); * *p*-value < 0.05 is significant.

**Table 2:** Outcome of chest compressions conducted between the intervention and control.

Outcome	Intervention (n = 20)	Control (n = 20)	*p*-Value *
CC depth, mm	52.1 (46.3–54.8)	47.1 (40.5–49.9)	0.021
Proportion of accurate-depth CCs, %	88.7 (30.0–99.1)	22.6 (0.0–58.5)	0.033
CC rate, counts/min	99.6 (99.5–100.0)	99.6 (99.6–99.8)	0.616
Proportion of complete chest decompression, %	100 (99.7–100.0)	100 (95.1-100)	0.306

Values are means (standard deviations), medians (interquartile range), or counts (proportion), and compared using a Wilcoxon’s signed rank sum test. Chest compression (CC) depth had the mean value of 3 min. The proportion of accurate-depth CCs was defined as the ratio of the number of CCs in which the depth was 5 to 6 cm to the total number of CCs. * *p*-value of less than 0.05 was considered a significant difference.

**Table 3:** Multivariate analysis of chest compression depth and the proportion of accurate-depth chest compressions.

Outcome	B	SE	VIF	*p*-Value **
* CC depth, mm				
Sex, male	8.88	2.21	1.33	0.019
BMI (kg/m^2^)	0.93	0.30	1.33	0.003
Feedback	6.11	1.66	1.00	<0.001
Proportion of accurate-depth CCs, %				
Sex, male	35.39	10.91	1.33	<0.001
BMI (kg/m^2^)	5.60	1.47	1.33	<0.001
Feedback	31.33	8.19	1.00	<0.001

B, standardized coefficient; SE, standard error; VIF, variance inflation factor; CC, chest compression; BMI, body mass index. * CC depth was shown as the mean value of 3 min. ** *p*-value < 0.05 is significant. A backward, stepwise, multivariate linear regression model was used. Age, sex, BMI, and feedback status were adjusted for each outcome; none of the regression models were statistically significant for either outcome (cardiac compression rate or the proportion of complete chest decompression).

## Data Availability

The data presented in this study are available on request from the corresponding author (J.L.).

## Supplementary Materials

The following are available online at https://www.mdpi.com/article/10.3390/ijerph18105408/s1, Figure S1: Bangor’s SUS score criteria and Table S1: Outcome of chest compressions conducted between the intervention and control according to sex.
