# Supplementary material for: Impact of a Smart-Ring-Based Feedback System on the Quality of Chest Compressions in Adult Cardiac Arrest: A Randomized Preliminary Study
Source: Int J Environ Res Public Health. 2021 May 19;18(10):5408. doi: 10.3390/ijerph18105408 (PMC8158714; doi:10.3390/ijerph18105408)
Supplement: Supplementary file 1 [file ijerph-18-05408-s001.zip › ijerph-1203621-supplementary.pdf]

Supplementary Material

Figure S1. Bangor's SUS score criteria.

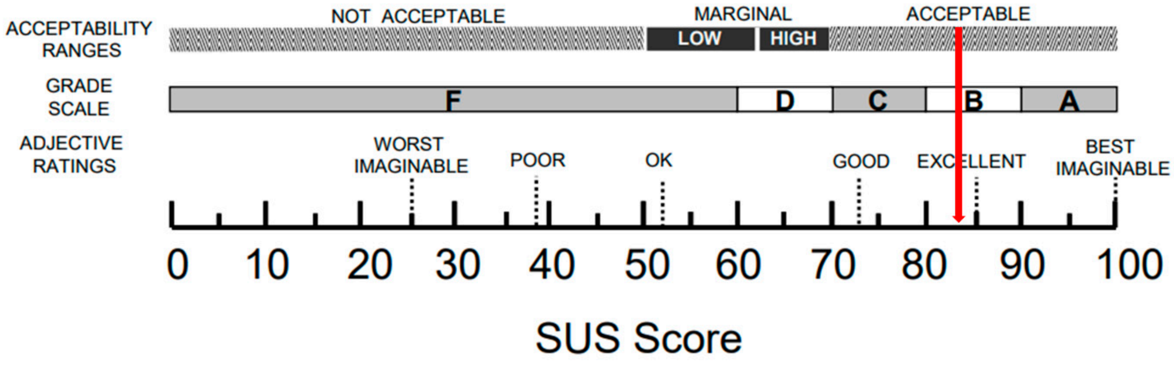

*SUS score of the smart ring in this study was 83.9 points, and it was marked with a red line.

Table S1. Outcome of chest compressions conducted between the intervention and control according to sex.

Sex	Intervention	Control	P value*
Male (n=15)			
CC depth, mm	52.6±4.6	48.4±6.1	0.026
Proportion of accurate CC depth, %	83.6±26.5	47.6±38.7	<0.001
CC rate, counts/min	99.2±2.0	99.5±1.3	0.668
Proportion of complete chest decompression, %	97.6±5.8	90.3±23.4	0.212
Female (n=5)			
CC depth, mm	44.0±6.1	32.4±7.2	0.032
Proportion of accurate CC depth, %	18.1±24.8	0.83±1.9	0.008
CC rate, counts/min	99.8±0.5	101.7±4.6	0.832
Proportion of complete chest decompression, %	100.0±0.0	100.0±0.0	-

Values are mean (SD), median [IQR], or number (proportion), and tested by the Mann-Whitney test. Proportion of accurate CC depth was defined as the ratio of the number of CC whose depth was between 5 and 6 cm to the total compression number for 2 min. CC, chest compression.

*p-value <0.05 is significant.
